# Kennedy's disease from India: An Indian Cohort with multisystemic manifestations

**DOI:** 10.1177/22143602251325795

**Published:** 2025-05-05

**Authors:** Saranya B Gomathy, William L Macken, Nimita Rani, Ayush Agarwal, Rakesh Singh, Megha Dhamne, Sruthi S Nair, Alisha Reyaz, Tanveer Ahmed, Ashwin Dalal, Mayandi Muthulakshmi, Lindsay Wilson, Asish Vijayaraghavan, ICGNMD Consortium, Rohit Bhatia, Robert D S Pitceathly, Kumarasamy Thangaraj, Mary M Reilly, Padma MV Srivastava, Michael G Hanna, Venugopalan Y Vishnu

**Affiliations:** 1 Department of Neurology, Jawaharlal Institute of Postgraduate Medical Education and Research, Puducherry, India; 2 Department of Neuromuscular Diseases, UCL Queen Square Institute of Neurology, London, UK; 3 NHS Highly Specialised Service for Rare Mitochondrial Disorders, Queen Square Centre for Neuromuscular Diseases, The National Hospital for Neurology and Neurosurgery, London, UK; 4 Department of Neurology, All India Institute of Medical Sciences, New Delhi, India; 5 Department of Neurology, Grant Govt. Medical College & Sir J. J. Group of Hospitals, Mumbai, India; 6 Department of Neurology, PD Hinduja Hospital & Medical Research Centre, Mumbai, India; 7 Department of Neurology, Sree Chitra Tirunal Institute for Medical Sciences and Technology, Thiruvananthapuram, India; 8 Diagnostics Division, Centre for DNA Fingerprinting and Diagnostics, Hyderabad, India; 9 International Centre for Genomic Medicine in Neuromuscular Diseases (ICGNMD) at UCL, London, UK; 10 Centre for Cellular and Molecular Biology, Hyderabad, India; 11 Department of Neurology, Paras Health, Gurgaon, Haryana, India

**Keywords:** Kennedy's disease, multisystem involvement, CAG repeats

## Abstract

**Background:**

Kennedy's disease (KD) is a rare, insidiously progressive lower motor neuron syndrome characterised by amyotrophy involving the appendicular or bulbar musculature of adult males in their fourth to fifth decade. There are no large series from the Indian subcontinent describing the clinical-genetic and laboratory spectrum of KD.

**Aim:**

To describe the clinical, electrophysiologic, metabolic and genetic profile of patients with KD.

**Methods:**

We conducted a retrospective review of ten genetically confirmed KD patients.

**Results:**

The mean age of the cohort was 47 years, with a mean age of onset of illness at 41.3 ± 9.9 years. The median duration of symptoms before presentation was 5 (3–12) years. The most common referral diagnosis was ALS. The majority presented with symmetric proximal limb weakness with bulbar symptoms and were found to have gynecomastia, lower motor neuron (LMN) facial weakness, and facial and lingual fasciculations. Electrophysiology revealed sensory neuropathy in five patients and chronic neurogenic changes consistent with anterior horn cell disease in all. Metabolic profile showed impaired glycemia, hyperlipidemia and evidence of non-alcoholic fatty liver disease in the majority. All had elevated serum creatine kinase. Genetic testing revealed a median of 46 CAG repeats. The phenotypes of our patients aligned with global data that is predominantly derived from participants of European ancestry.

**Conclusion:**

We describe a series of patients with KD from India with significant multisystemic involvement.

## Introduction

Kennedy's disease (KD), also known as Spinal Bulbar Muscular Atrophy (SBMA) is a rare, lower motor neuron disorder characterised by weakness, wasting and fasciculations involving appendicular or bulbar muscles, and affecting adult males in their fourth to fifth decade of life.^1,2^ Studies based primarily on patients of European ancestry estimate its occurrence as 1 in 30,000 men.^3^ It occurs due to cytosine-adenine-guanine (CAG) repeat expansion in exon 1 of the androgen receptor gene on the X-chromosome.^4^ In healthy, unaffected males, the size of this region ranges from 12 to 30 CAG repeats, while in SBMA patients, the number of repeats is 38 or more.^5,6^ The pathophysiology underpinning this disease is toxic accumulation of androgen receptor aggregates in the cell nucleus leading to cell death and androgen insensitivity.^7,8^ KD usually manifests with progressive proximal lower limb weakness with wasting and later progresses to involve the distal and bulbar muscles. Fasciculations, especially in the face and perioral region, and hyporeflexia are generally seen.^2,6^ Other manifestations include muscle cramps, sensory neuropathy, fatigability and postural tremors, which can antedate the muscle weakness. KD is considered to be a multisystem disorder with metabolic and endocrine derangements in the form of impaired glucose tolerance, thyroid abnormalities, dyslipidaemia, sexual dysfunction and gynecomastia.^9–11^ Variable expressivity may occur within a family including the age of onset, symptoms encountered, and disease severity.^9^ Various studies have shown that CAG repeat length expansion size affects the severity of disease and inversely correlates with age of onset.^5,12,13^ Additional polygenic, stochastic and environmental factors also may play a role. The differential diagnosis includes Amyotrophic Lateral Sclerosis (ALS), in view of bulbar weakness and fasciculations, and limb girdle muscular dystrophy, as raised CK with limb weakness is also seen in KD. However, disease progression, and multisystemic involvement can differentiate KD from the above two conditions. Genetic testing finally clinches the diagnosis, but standard tests used in high income settings are not routinely available in Indian scenario without great cost to the patient.

KD has important variation between populations, with the highest prevalence in the prairie provinces of Western Canada due to a genetic founder effect.^14,15^ Similarly, it has a very high prevalence in the Vasa region of Western Finland, and is twice more common compared to ALS in this region.^16^ In Asia, higher prevalence of KD is seen in Japan.^5,17^ The true prevalence of KD in the Indian subcontinent is unknown. There are only nine genetically confirmed cases of Kennedy's disease reported from India.^18–20^ This largest case series from India comprising ten KD patients, thereby doubling published data, describes the various phenotypic manifestations and molecular characteristics of Kennedy's disease as well as some case-specific observations. Even though there is currently a lack of effective therapy, a genetic diagnosis is of great importance for prognostication and genetic counselling in both patients and relatives.

## Methods

Patients’ data was retrospectively collected from the databases of four tertiary care hospitals between 2015–2023, which included All India Institute of Medical Sciences Comprehensive Neuromuscular Disorders Centre (AIIMS-CNMD), AIIMS New Delhi; Grant Govt. Medical College & Sir J. J. Group of Hospitals, Mumbai; PD Hinduja Hospital, Mumbai and Sree Chitra Tirunal Institute for Medical Sciences and Technology, Thiruvananthapuram. At AIIMS-CNMD, participants provided informed consent to participate in the International Centre for Genomic Medicine in Neuromuscular Diseases (ICGNMD) study for phenotypic, clinical and family history data collection and research testing. In other centres, individual written consent was taken from the patients. Inclusion criteria were insidious onset, gradually progressive LMN weakness with wasting and/or fasciculations, bulbar involvement, endocrine involvement and confirmed by molecular genetic testing. Patients who had cervical or brain magnetic resonance imaging (MRI) lesions were excluded. The screening criteria were insidious onset, gradually progressive LMN weakness with wasting and/or fasciculations with or without bulbar involvement. Baseline demographic and clinical features, and laboratory investigations were collected from hospital records. All patients underwent deep phenotyping, nerve conduction studies and electromyography, MRI of the brain and spine, along with blood investigations including creatine kinase (CK), liver and renal functions, Follicle Stimulating Hormone (FSH), Luteinizing Hormone (LH), estradiol, testosterone, fasting lipid profile, fasting blood sugar levels. The mutation confirmation was done in venous blood by short polymerase chain reaction (PCR) using fluorescently labelled primers flanking the CAG repeat region of the androgen receptor (AR) gene (Forward primer-TCC AGAATCTGTTCCAGAGCGTGC and reverse primer-GCTGTGAAGGTTGCTGTTCCTCAT). The stock primers were maintained at a concentration of 100 pmoles per microliter. To make the working primer solution, each primer stock was 10 times diluted in Mili Q water to make a final volume of 50 microliter. The stock and the working primer solutions were kept refrigerated at −20°C until use. For genotyping, sample preparation was done in a 96-well plate and then denatured at 96°C for 4 min followed by quick chilling in ice and loaded on to ABI3730 Genetic analyzer. Samples were analyzed by Genemarker v2.2.0 software and reporting were done based on the ACMG guidelines. The mutation analysis was done at the Diagnostic Division of the Centre for DNA fingerprinting and Diagnostics, Ministry of Science and Technology, Government of India, Hyderabad.

## Results

### Clinical features

A total of twenty-three individuals with suspected Kennedy's disease were tested. Six probands were found to have normal number of CAG repeats. Ten probands and one affected relative had genetically confirmed Kennedy's disease. All patients belonged to Asian ethnicity and all of them were male patients. The mean age of the cohort comprising 10 patients was 47 years, ranging between 36 to 61 years. The mean age of onset of illness was 41.3 ± 9.9 at years of age. The median duration of symptoms prior to presentation was 5 (3–12) years. The most common referral diagnosis was ALS in six patients followed by Limb girdle muscular dystrophy in four patients.

**Figure 1.**
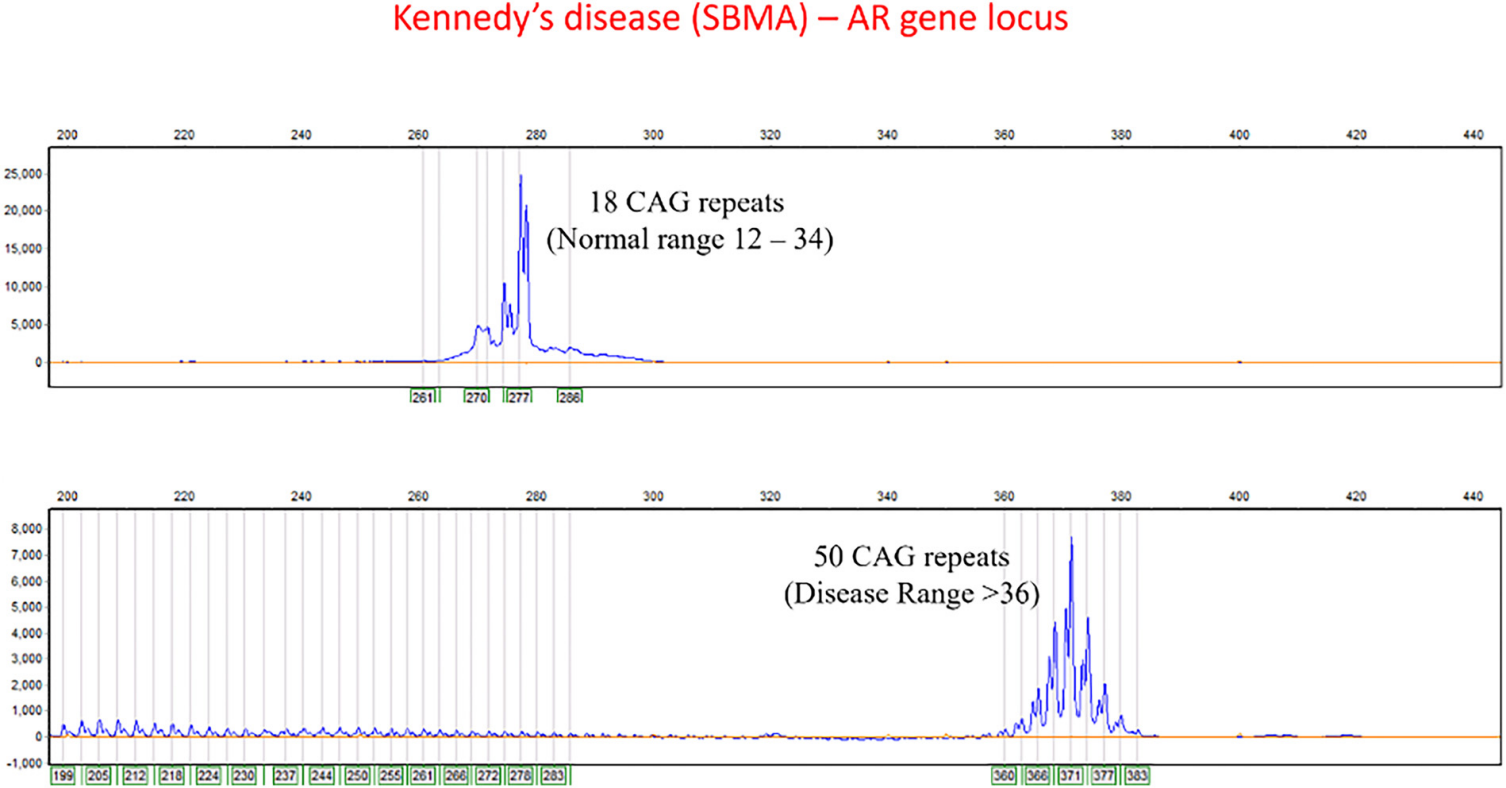
Representative figure of Kennedy's disease repeats.

Five (5/10) patients presented with complaints of symmetric proximal weakness of bilateral upper and lower limbs with bulbar involvement; three (2/10) had distal limb asymmetric amyotrophy with bulbar involvement, and one had pure proximal upper and lower limb without bulbar involvement. Three (3/10) patients presented with excessive fatigability and muscle cramps. Flaccid dysarthria and dysphagia were reported by six and three patients respectively; neck weakness was present in one patient. Gynecomastia, lingual fasciculations with atrophy and bifacial LMN weakness were universal findings while 8/10 patients had facial and perioral fasciculations on examination. Erectile dysfunction was reported by one patient. Polyminimyoclonus was present in three, reduced deep tendon reflexes were noted in nine, and none had exaggerated deep tendon reflexes. One patient had diminished perception of vibratory sensation in lower limbs. One had subtle cerebellar findings in the form of gaze evoked nystagmus and impaired tandem gait. A strong family history of similar illness in males was reported by 7/10 patients. The clinical features are summarized in Table 1. The representative figure of KD repeats is given as Figure 1.

**Table 1.** Clinical characteristics of KD patients.

Patient characteristics	Patient 1	Patient 2	Patient 3	Patient 4	Patient 5	Patient 6	Patient 7	Patient 8	Patient 9	Patient 10
Age of onset, years	34	42	33	30	38	29	47	58	45	57
Age at presentation, years	38	48	45	36	46	39	50	61	48	61
Family history	+(brother)	+(brother)	+(brother)	+(maternal cousin brother)	+(three brothers have similar disease)	Nil	Nil	+(brother)	+ (two maternal uncles died at the age of 60 years due to similar illness)	Nil
Presenting symptom	Fatigability, difficulty in chewing and swallowing	Distal UL and proximal LL weakness associated with diffuse fasciculations and cramps	Proximal UL and LL weakness associated with dysarthria and dysphagia	Proximal UL and LL weakness with bifacial weakness	Proximal UL and LL weakness with bifacial and neck weakness	Proximal UL fatigability and facial fasciculations	Asymmetric weakness of distal UL followed by dysphagia and dysarthria; he also had erectile dysfunction	Bulbar symptoms with proximal UL and LL weakness; noticed after he recovered from COVID infection	Symmetric proximal LL weakness with bilateral UL tremors	Asymmetric onset proximal LL weakness with amyotrophy, distal UL weakness and wasting, bifacial weakness, dysarthria
Gynaecomastia	+	+	+	+	+	+	+	+	+	+
Polyminimyoclonus	-	-	-	+	-	-	+	-	+	+
Limb Fasciculations	-	+	-	+	+	-	+	-	-	-
Tongue atrophy	+	+	+	+	+	+	+	+	+	+
Tongue fasciculations	+	+	+	+	+	+	+	+	+	+
Jaw weakness	+	-	-	-	-	-	-	-	-	-
Facial weakness	+	+	+	+	+	+	+	+	+	+
Facial and perioral fasciculations	+	+	+	+	+	+	+	-	+	+
Bulbar involvement	+	-	+	+	+	-	+	+	+	+
Deep tendon reflexes	Absent in all 4 limbs	Absent ankle jerks and 2 + elsewhere	Hyporeflexia in LL and areflexia in UL	Generalised areflexia	Generalised hyporeflexia	Normal in all the 4 limbs	Generalised areflexia	Generalised areflexia	Generalised areflexia	Generalised hyporeflexia
Sensory findings	Absent	Absent	Absent	Absent	Absent	Absent	Reduced vibration perception in lower limbs	Absent	Absent	Absent
Erectile dysfunction	No	No	No	No	No	No	Yes	No	No	No

### Electrophysiological findings and metabolic profile

Nerve conduction studies revealed sensory axonal polyneuropathy in four patients, sensorimotor axonal neuropathy in one and electromyography (EMG) showed chronic axonopathic process involving cervical, thoracic, lumbosacral and bulbar segments in eight patients, and cervical and lumbosacral involvement alone in two patients. Repetitive nerve stimulation test was performed in two patients and it did not show any decremental response. None of the patients had heart disease or demonstrated ECG abnormalities suggestive of Brugada syndrome.

Six (5/10) had impaired fasting glucose, five manifested hypertriglyceridemia and seven had elevated LDL levels. Seven patients (7/10) had transaminitis and one had manifest hypothyroidism. Luteinizing hormone (LH) and estradiol levels were high in 4/10 and 6/10 patients respectively. CK was elevated in all, and patient 3 had CK elevation 24 times the upper limit of normal. Details are summarized in Table 2.

**Table 2.** Electrophysiology, metabolic and genetic profile of KD patients.

Patient characteristics	Patient 1	Patient 2	Patient 3	Patient 4	Patient 5	Patient 6	Patient 7	Patient 8	Patient 9	Patient 10
Nerve conduction study	Sensory axonal neuropathy affecting all 4 limbs	Normal	Normal	Sensory axonal neuropathy affecting all 4 limbs	Normal	Normal	SNAPs absent from both upper and lower limb nerves	Sensory axonal neuropathy affecting all 4 limbs	Normal	Sensorimotor axonal neuropathy affecting all 4 limbs
EMG	Neurogenic involvement in bulbar, cervical and lumbosacral segments	Neurogenic involvement in bulbar, cervical and lumbosacral segments	Neurogenic involvement in bulbar, cervical and lumbosacral Segments	Neurogenic involvement in bulbar, cervical and lumbosacral segments	Neurogenic involvement in bulbar, cervical and lumbosacral Segments	Neurogenic involvement in cervical and lumbosacral segments	Neurogenic involvement in bulbar, cervical and lumbosacral segments	Neurogenic involvement in bulbar, cervical and lumbosacral segments	Neurogenic involvement in cervical and lumbosacral segments	Neurogenic involvement in bulbar, cervical and lumbosacral segments
ECG	Normal	Normal	Normal	Normal	Normal	Normal	Normal	Normal	Normal	Not done
MRI brain + Spine	Normal	Normal	Normal	Normal	Normal	Normal	Normal	Normal	Not done	Not done, PET-CT normal
Fasting blood sugar, mg%	140	150	155	118	121	105	NA	NA	90	200
Total cholesterol, mg% (<200 mg%)	207	195	194	175	172	189	NA	NA	199	298
Triglycerides, mg% (<150 mg%)	179	84	232	151	115	160	NA	NA	50	520
High density lipoprotein (HDL), mg%	36	84	41	55	43	43	NA	NA	45	39
Low density lipoprotein (LDL), mg% (<100 mg%)	134	122	106	90	107	114	NA	NA	144	170
Thyroid function	Normal	Hypothyroidism	Normal	Normal	Normal	Normal	Normal	Normal	Normal	Normal
Alanine aminotransferase (ALT), IU/L	**119**	**99**	**111**	47	34	**218**	NA	NA	**102**	**59**
Aspartate aminotransferase (AST), IU/L	**93**	**81**	**100**	**72**	45	**123**	NA	NA	**97**	38
Follicle stimulating hormone (FSH), U/L (1.5–12.4)	5.29	4.8	6.66	3.69	6.2	3.4	NA	NA	2.67	5.3
Luteinizing hormone (LH), IU/mL (1.7–8.6)	6.98	9.2	11.22	5.85	11.8	14.3	NA	NA	6.42	4.7
Estradiol, pg/ml (10–50)	50.26	54.4	85.13	48.93	92	92	NA	NA	NA	NA
Testosterone, ng/ml (2.8–8)	5.19	3.8	4.22	4.58	4.8	4.8	NA	NA	8.41	496
Creatine kinase (CK), U/L (55–170)	418 at 37 years; 688 at 37 years; 829 at 38 years; 704 at 38 years	812 at 48 years	4027 at 45 years; 2923 at 45 years; 3241 at 44 years	896 at 35 years	542 at 42 years	1029 at 35 years; 1232 at 36 years	NA	NA	1850 at 48 years	331 at 61 years
No. of CAG repeats (Normal 9–36)	46 (Brother-19 repeats)	47 (Brother-47 repeats)	47	48	50	49	46	42	44 ± 3	38 ± 3

The individual cases are described in detail.

#### Case 1

A 38-year-old male, presented with a four-year history of difficulty in chewing and dropping of jaw, dysphagia, flaccid dysarthria and fatigability of upper limbs with no limb weakness, sensory complaints, or bladder involvement. He had gynecomastia, bifacial LMN weakness, facial and perioral fasciculations, jaw weakness with absent jaw jerk, atrophic tongue with fasciculations, and generalized hyporeflexia with MRC grade 5/5 power in all four limbs. Electrophysiologic study revealed a sensory axonal neuropathy with neurogenic changes in cervical, bulbar and lumbosacral segments. Creatine kinase (CK) levels ranged between 418–829 U/L. He had elevated fasting blood sugar levels and hypercholesterolemia. Serum estradiol was elevated with normal testosterone, FSH and LH levels. Genetic study uncovered 46 CAG repeats. His brother also had gynecomastia however his genetic analysis showed 19 CAG repeats, which was in the normal range.

#### Case 2

A 48-year-old male with onset of disease at the age of 42 years with a positive family history of similar illness in a brother, presented with fasciculations predominantly over the face and both upper and lower limbs in addition to proximal lower limb and distal upper limb weakness with cramps involving both lower limbs. He had gynecomastia with bifacial LMN weakness, and facial and tongue fasciculations. Power was MRC grade 4/5 in both distal upper limbs and proximal lower limbs. Deep tendon reflexes were normal in both upper limbs and knee with absent ankle jerks. Nerve conduction study was normal and EMG showed neurogenic involvement of cervical, bulbar and lumbosacral segments. Serum CK was elevated (812 U/L) along with elevated LH and estradiol, and fasting blood glucose. Molecular analysis showed 47 CAG repeats. The brother (aged 40 years) who had similar clinical features, had 48 CAG repeats.

#### Case 3

A 45-year-old male with onset of illness at 33 years of age and a reported similar history in a brother, had bilateral symmetric proximal upper and lower limb weakness with dysphagia, and hypernasal speech. He had gynecomastia, bifacial weakness with facial, perioral and tongue fasciculations, generalized hypotonia, hyporeflexia and MRC grade 3/5 power involving the proximal upper and lower limb musculature associated with amyotrophy. Serum CK was highly elevated (3923 IU/L) and he had diabetes, hypertriglyceridemia and transaminitis, along with elevated LH and estradiol. Axonopathic process was noted involving three segments. He had 47 CAG repeats; his brother could not be tested.

#### Case 4

A 36-year-old male presented with a six-year history of progressive weakness of both upper and lower limbs with facial weakness and fasciculations involving all 4 limbs. He had gynecomastia, facial/perioral fasciculations, tongue atrophy with fasciculations, MRC grade 4/5 power in both proximal upper limbs and grade 3/5 power in bilateral lower limbs with generalized areflexia and polyminimyoclonus. Sensory axonal neuropathy and neurogenic changes involving three segments were noted on EMG. Creatine kinase was 896 U/L. His hormonal profile was normal and he had 48 CAG repeats.

#### Case 5

A 46-year-old male presented with complaints of gradually progressive proximal weakness of all 4 limbs with fasciculations, tongue atrophy and facial weakness from the age of 38 years. Proximal upper and lower limb power was MRC grade 4/5. He had gynecomastia, neck weakness, tongue fasciculations and generalized hyporeflexia. Sensory nerve action potential amplitudes were normal in all tested nerves and there was neurogenic involvement of three myotomes. Creatine kinase was 542 U/L. Hormonal study revealed elevated LH and estradiol levels. He had 50 CAG repeats.

#### Case 6

A 39-year-old male presented with a 10-year history of bilateral proximal upper limb fatigability associated with twitching of muscles over the face and tongue fasciculations. He had gynecomastia with bifacial LMN weakness, facial and perioral fasciculations, and atrophic tongue with florid fasciculations. Tone was normal, with MRC grade 5/5 power in all the four limbs with normal reflexes. Creatine kinase was elevated at 1232 U/L, and he had transaminitis, elevated LH and estradiol. On genetic testing, he had 49 CAG repeats and three segments involved in EMG.

#### Case 7

A 50-year-old male presented with a 3-year history of asymmetric onset distal amyotrophy of both upper limbs with 6 months’ history of slurring of speech and dysphagia, along with flickering of muscles around his mouth and erectile dysfunction. On examination, he had gynecomastia and asymmetric hand weakness, more on right with bifacial weakness, polyminimyoclonus, tongue fasciculations, areflexia and vibration impairment at bilateral great toes. Nerve conduction study revealed absent SNAPs and EMG showed neurogenic changes in three segments. Fasting blood sugar, lipid profile and liver functions were within normal limits. Genetic testing revealed 46 CAG repeats.

#### Case 8

A 61-year-old male with no comorbidities presented with a 3-year history of progressive dysphagia and dysarthria associated with proximal weakness of both upper limbs. He noticed it following COVID-19 infection in 2020. Examination showed flaccid dysarthria with mild LMN bifacial weakness, spastic tongue with fasciculations, MRC grade 4/5 power in bilateral proximal upper extremities, and generalized areflexia. Sensory axonal neuropathy with neurogenic involvement in 3 segments were present. Genetic testing uncovered 42 CAG repeats. There was positive family history of a neuromuscular disorder in an elder brother who succumbed to the illness.

#### Case 9

A 48-year-old male, hypertensive, manifested with symmetric proximal lower limb weakness of three years’ duration and bilateral upper limb tremors. He was operated for gynecomastia in his twenties. Two maternal uncles had history of proximal lower limb weakness with fasciculations, with onset after 60 years of age, and they became bedridden 10 years into the illness. On examination, he had left gynecomastia, gaze evoked nystagmus, right sided facial fasciculations, bifacial weakness and polyminimyoclonus of bilateral upper limbs. Power was MRC grade 4+/5 in bilateral proximal hip regions with 5/5 power elsewhere with sluggish deep tendon reflexes. Gait was broad-based. Creatine kinase was 1850 U/L. Nerve conduction study was normal, and EMG showed neurogenic changes in cervical and lumbosacral myotomes. He had transaminitis and ultrasonography of abdomen revealed Grade 3 fatty liver. MRI brain was normal. His molecular testing revealed 44 ± 3 CAG repeats.

#### Case 10

A 61-year-old male, diabetic for 20 years came with 4 years history of asymmetric onset weakness and wasting involving right hand and proximal bilateral lower limb weakness and bifacial weakness. He had gynecomastia, facial fasciculations, polyminimyoclonus, bifacial LMN weakness, tongue atrophy and fasciculations, deviation of uvula to the right, generalized hypotonia with wasting of right thenar muscles, bilateral first dorsal interossei, bilateral forearm muscles, and bilateral quadriceps. Power was MRC grade 4/5 in bilateral proximal upper limbs, with wrist extension weaker on the right side compared to the left, and proximal bilateral lower limb power was 4-/5 with generalized hyporeflexia. Creatine kinase was 337 U/L. NCS showed asymmetric sensorimotor axonal polyneuropathy with neurogenic involvement of 3 segments on EMG, and he had 38 ± 3 CAG repeats on molecular testing.

Table 1 describes the demographic characteristics and clinical presentation of the KD patients. Table 2 details the electrophysiological, metabolic, hormonal and genetic profile of these patients.

## Discussion

This is the largest case series from the Indian subcontinent describing clinical, neurophysiology, endocrine and genetic characteristics of ten KD patients. The mean age of the disease onset in our series was 47 years, in line with previous Indian and European studies.^5,20–22^ Patients presented with typical features of Kennedy's disease with atrophy and weakness of the proximal musculature and bulbar involvement. Previous international studies demonstrated that non-motor symptoms like fatigue, cramps and muscle pain may antedate muscle weakness by months to years; however, the three out of ten patients in this series reported fatigue and muscle cramps, starting at the same time as motor weakness.^23,24^ Only one patient had preceding non-motor symptoms. Fatigue is due to the chronic neurogenic process that causes defective motor end plates and thereby defective myoneural junction transmission.^25^ Universal findings in our cohort were gynecomastia and tongue fasciculations, which are considered as clinical pointers in KD; gynecomastia occurs due to androgen insensitivity, and has been very frequently reported in various cohorts.^6,12,21^ One patient had predominant distal upper limb postural tremors as a presenting symptom, similar to the observations by Atsuta et al.^5^ Rhodes et al. described the clinical features of 57 patients with KD, and found tremor in 23% of patients.^6^ Tremor can precede muscle weakness by several years; a high index of suspicion of KD should be therefore kept in men who present with distal upper limb tremors and features of androgen insensitivity. Most patients had bifacial LMN weakness, and facial and perioral fasciculations; these findings are considered the hallmark findings in KD. All had diminished-to-absent deep tendon reflexes and none had exaggerated reflexes or upper motor neuron signs, which is in line with previous observations.^5,6,20^

A unique finding noted in patient 9 was presence of nystagmus and wide based gait. Cerebellar signs have not been previously described with KD and this case adds to the phenotypic spectrum of KD. Whole exome sequencing could not be performed. Morphometric studies have demonstrated cerebellar white matter involvement in patients with KD and this may explain the subtle findings in our patient.^26^ But we cannot conclusively rule out a co-existing cerebellar disease due to lack of further evaluation.

Sensory symptoms are rarely reported by patients with KD; however, abnormalities on sensory nerve conduction in the form of reduced SNAP amplitudes are very frequent; five patients in this series demonstrated SNAP amplitude reduction which indicates chronic axonal abnormalities in these patients.^5,23^ All patients had chronic neurogenic involvement on EMG which is the expected finding in KD.

Serum creatine kinase (CK) was elevated in all patients, similar to previous studies.^5,27^ Creatine kinase elevation up to 8 times the upper limit of normal has been reported with KD; the reason is not well understood.^27^ It is postulated that CK elevation in KD occurs as a result of a myopathic process due to failure of activation of satellite cells due to the mutant androgen receptor protein accumulation.^28^ The fact that hyperCKemia is higher in KD compared to other motor neuronopathies like Amyotrophic lateral sclerosis, indicates that this disease might also involve muscle fibres apart from motor neurons.^28–30^

Six patients in this series had diabetes mellitus, five had hypertriglyceridemia and seven patients had asymptomatic transaminitis, similar to previous studies.^6,12,31,32^ Mechanisms involved in the metabolic alterations of KD are still not well understood. Insulin resistance is implicated in causing both metabolic syndrome and non-alcoholic fatty liver disease (NAFLD). Testosterone and androgen receptor play a role in insulin signalling; and in the face of low testosterone levels and defective androgen receptor, metabolic syndrome and NAFLD ensue.^33^ Insulin sensitivity and consequent metabolic syndrome is also influenced by the CAG repeat length polymorphism.^34^ The median CAG repeat length was comparable to that in other ethnic cohorts.^5,6,20,35^

Barriers to testing, particularly in resource-limited settings like certain Indian states, might include limited access to specialized diagnostic facilities, lack of awareness among healthcare providers, and the associated costs of genetic testing. Exploring these potential barriers further could provide valuable context for understanding regional differences in diagnosis rates. Additionally, while a genuinely low prevalence of KD in certain populations cannot be ruled out, it would be worthwhile to examine whether such data reflect underdiagnosis or misclassification of symptoms with other motor neuron disorders.

## Conclusion

We analysed ten patients with KD and confirmed the multisystemic involvement of the disease process, and noted a unique finding of cerebellar ataxia in one patient.
